# Evaluation of the response to electric pulp testing in multiple sclerosis patients without a history of trigeminal neuralgia: a case-control study

**DOI:** 10.1186/s12883-021-02416-0

**Published:** 2021-10-20

**Authors:** Fatemeh Owlia, Nazanin Mahmoudzade, Jalil Modaresi, Marzieh Abutorabi Zarchi

**Affiliations:** 1 Department of Oral and Maxillofacial Medicine, Social Determinants of Oral Health Research Center, School of Dentistry, Shahid Sadoughi University of Medical Sciences and Health Services, Yazd, Islamic Republic of Iran; 2 School of Dentistry, Shahid Sadoughi University of Medical Sciences and Health Services, Yazd, Islamic Republic of Iran; 3 Department of Endodontics, School of Dentistry, Shahid Sadoughi University of Medical Sciences and Health Services, Yazd, Islamic Republic of Iran; 4 Department of Neurology, Shahid Sadoughi University of Medical Sciences and Health Services, Yazd, Islamic Republic of Iran

**Keywords:** Electric pulp test, Multiple sclerosis, Tooth pulp, Diagnosis, Dental stimulation

## Abstract

**Background:**

The importance of evaluating the pulpal threshold to electrical stimulation, as a side effect of probable neuropathy in Multiple Sclerosis (MS) patients is a novel issue. This study aimed to investigate electrical pulp test thresholds in MS patients without a history of trigeminal neuralgia compared to healthy individuals.

**Methods:**

Sixty-nine maxillary central incisors, belonging to 34 relapsing-remitting MS patients, and 35 healthy individuals were included in this survey. The MS patients matched for intended variables, were 22–50 years old, had a more than 1-year history of MS, no history of trigeminal neuralgia and/or other neuropathy. The electric pulp sensibility test was performed on all samples. Electric pulp testing (EPT) results were recorded based on the pulp tester^’^s grade that evoked a response. Data were analyzed with paired T-test, Mann-Whitney test, and Spearman correlation (*P* < 0.05).

**Results:**

According to the results of this study, the mean values of response to EPT were 1.2 ± 0.5 and 1.8 ± 0.5 in MS patients and healthy individuals, respectively. The pulpal response to EPT between the two groups was significantly different (*P* < 0.0001).

**Conclusions:**

MS patients showed a significantly reduced response to the electric pulp test in their maxillary central incisors in comparison to matched healthy persons.

## Background

Multiple sclerosis (MS) is the most commonly reported neurodegenerative disorder in the central nervous system (CNS). There is an increasing prevalence and incidence of it in the U.S. and the world. Migratory influence and environmental or genetic effects remain as main risk factors of the frequency of MS worldwide [1]. Peripheral sensory neuropathy is one of the popular complications of MS [2]. In addition to peripheral neuropathy, central involvement could occur in MS. Recent evidence in literature have shown that transcranial direct current stimulation (tDCS) as a non-invasive brain stimulation technique, may reduce or relieve chronic pain [3]. Patients often experience sensory impairments such as paresthesia and hyperesthesia [4]. Trigeminal nerve is one of the nerves that may be the most commonly involved [5]. Alveolar branches of trigeminal nerve innervate teeth. The appropriate scale is measuring the electrical stimulation threshold of teeth as a part of the trigeminal nerve evaluation [6]. MS as a demyelinating disease can also influence the function of the myelinated nerves and impair the transmission of sensory signals. The sensory nerves stimulated by electric pulp test (EPT) are A-fibers, located in the pulp. The different conditions may lead to disrupting EPT readout [7]. EPT as a non-invasive, standardized, reliable, reproducible, and easily-performed method, which provides qualitative sensory manifestations of dental pulp, was used in this study [8].. Study on sensory nerve of neuropathic disorders such as diabetes, celiac and MS has been the focus of attention since many years ago [9, 10]. However, study on tooth somatosensory evoked potentials usually ignored. Considering the lack of studies in this regard, this survey was designed as a first step for designing future experiments. This study was designed to determine the response of dental pulp to EPT of MS patients compared to healthy individuals.

## Methods

### Sample size calculation

To reach the satisfying level for significant results with regard to statistical formula, at least 30 persons should be studied in each group in this case-control study. The following parameters were employed: test power of 80%, standard error of 5%, and standard deviation of pulp test (SD = 1). In the test power of study, type I and type II errors were considered. The participants were divided into 2 groups of MS patients and healthy individuals.

### Inclusion criteria

According to the study design, out of 45 relapsing-remitting MS patients with normal nerve conduction parameters, no symptoms of trigeminal neuralgia in biography and no evidence of sensory/motor involvement in trigeminal nerve who fulfilled the inclusion criteria, 34 patients (13 men and 21women) with an age range of 22 to 50 years were randomly selected to participate in this Case-Control study. The patients had been referred to a specialized registered center for MS patients in Yazd, Iran. All of them were clinically on controlled status in spite of different Disease-Modifying Treatment (DMT). At least 1 year must be elapsed from the diagnosis and they ought to have no history of drug abuse. A very detailed history of symptoms, and physical examination, emphasizing sensory assessment was conducted. Patients who suffered from other diseases and nutritional or metabolic disorders, and those who were under medication that could affect the central or peripheral nervous system and uncontrolled and handicapped patients did not enter the study. History of taking tricyclic antidepressants and antihypertensive medication during the last 3 months and taking analgesics during 48 h before the sensibility tests were considered, too.

For the Control group, 35 healthy persons (13 men and 22 women) aged between 20 and 50 years were referred to the Department of Oral Medicine and Endodontics in School of Dentistry, Yazd Shahid Sadoughi University of Medical Sciences, with an age range of 20–50 years. They had no history of systemic disease or taking analgesics during 48 h when they were recruited to the study.

All the experimental procedures in the present study were approved by the Ethics Committee in Research at Shahid Sadoughi University of Medical Sciences, Yazd (IR.SSU.REC.1396.194). Informed written consent was obtained from each person before initiating the study. All methods were performed in accordance with the relevant guidelines and regulations.

Sensibility tests were applied to the maxillary intact central teeth without any restoration, caries lesion, periodontal problem, sensitivity to percussion, history of trauma, and orthodontic treatment. All participants were acquainted with the procedure before the beginning of the experiment and signed the informed written consent. All participants filled the HAD (Hospital Anxiety and Depression) scale questionnaire [11] to confirm a matched condition of anxiety. Cotton rolls were inserted above the tooth to isolate it from the upper lip. Teeth were dried using cotton gauze without air blasts. A toothpaste (Crest, New York, USA) was applied on the buccal surface of the maxillary central incisors as an interface media by the gloved tester. Electrical stimulation was supplied by the EPT device (Gentle-pulse, Parkell, USA). The probe was placed on the one-third incisal edge of the labial surface and the lip clip of the pulp tester was placed in the individual’s mouth. The number indicating the pulp tester current was recorded as the patients’ responses. Examination procedures were performed by the same operator and same EPT unit in accordance with manufacturers^,^ instructions. Testing of each tooth started after contact of the electrode tip with the tooth surface and terminated when the persons raised their hands to show sensing the first sensation (tingling). The electric pulp sensibility test responses in MS patients and healthy persons were recorded and compared.

### Statistical analysis

The data were collected and categorized in terms of subjects, duration of MS, and tooth responses. Data were analyzed using SPSS17 (SPSS17; Chicago, IL, USA). Results were expressed as mean and standard deviation. Non-parametric tests were used because not all data passed the Kolmogorov-Smirnov normality test. Mann-Whitney test was applied to compare the electrical stimulation threshold of maxillary central incisors between MS patients and the Control group. Paired T-test was used to assess the significance of the difference in age between the two groups. Spearman correlation was used to discover the relationship between the duration of MS and the mean value of the sensibility threshold (*P* < 0.05).

## Results

All participants completed the study. The Mean ± SD of age in MS patients was 32.7 ± 6.6 years with a range of 22 to 50 years. This value was 33.2 ± 8.5 years in the control group with a range of 20 to 50 years. According to Table 1, based on the T-test, the two groups were similar in age (*P* = 0.8) (Table 1). Also, 21 (61.8%) of MS patients were female, whereas 13 (38.2%) patients were male as shown in Table 1. This ratio was almost similar to the Control group. Mean threshold values of EPT in two groups were evaluated with regard to gender. The mean value of sensibility threshold in the Control group for male and female individuals was recorded as 1.8 ± 0.3 (minimum = 1 and maximum = 2) and 1.7 ± 0.6 (minimum = 1 and maximum = 3), respectively.

**Table 1:** Demographic data of studied groups

**Groups**	**Number**	**Age (Mean ± SD)**	**Male** **N (%)**	**Female** **N (%)**
Healthy subjects	35	33.2 ± 8.5	13 (37.1)	22 (62.9)
MS patients	34	32.7 ± 6.6	13 (38.2)	21 (61.8)

T-test

*P* = 0.08

**Fig. 1:**
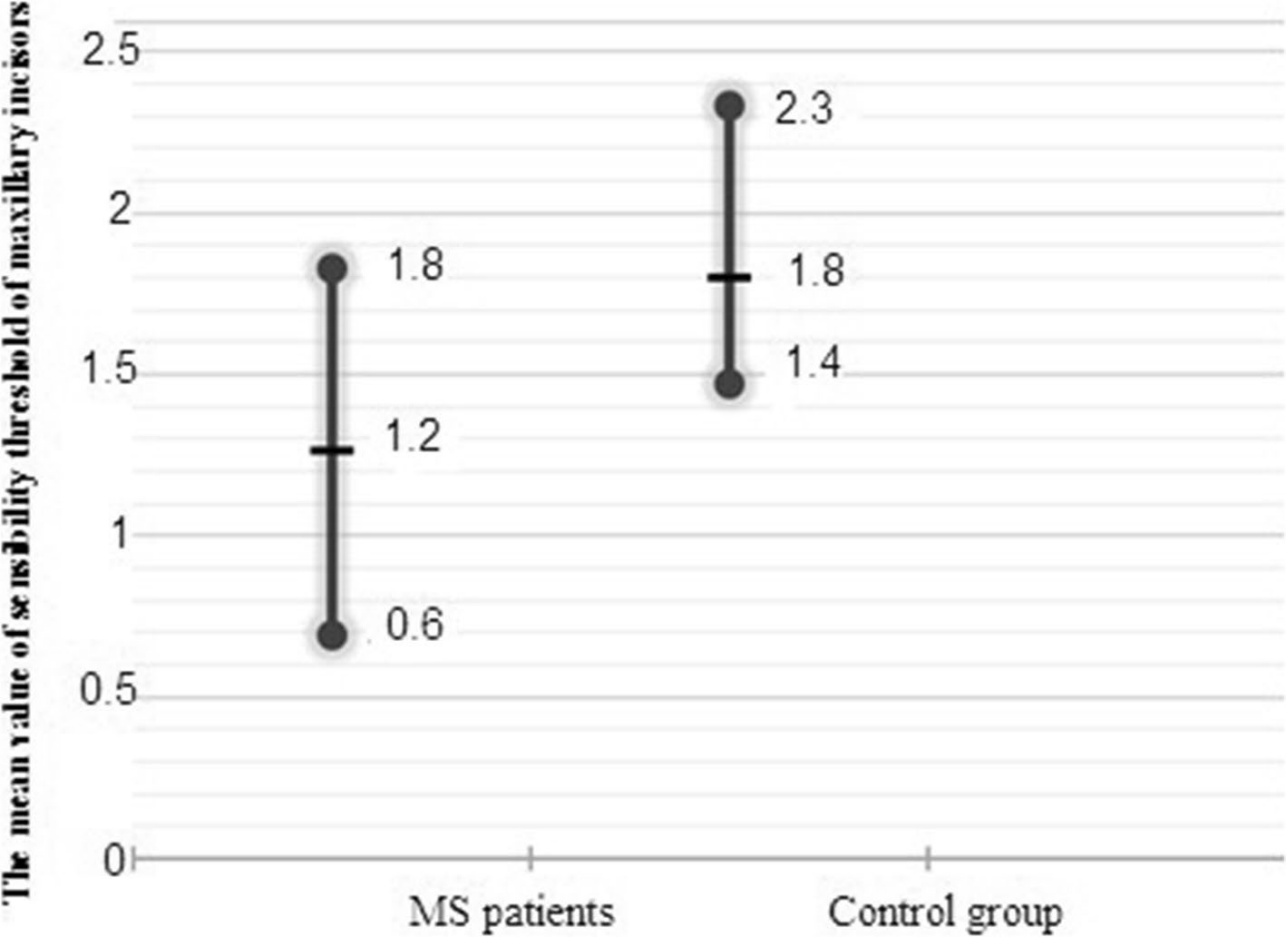
Comparison of mean threshold value of EPT in MS patients with Control group

Tooth responses to the EPT sensibility tests in MS patients was statistically lower than Control group. (*P* = 0.0001) (Table 2 and Fig. 1).

**Table 2:** Comparison of the response to electric pulp testing in MS patients and healthy subjects

**Groups**	**Number**	**Mean ± SD**
Control group	35	1.8 ± 0.5
MS patients	34	1.2 ± 0.5

Mann-Whitney Test

*P* = 0.0001

The average response to the EPT of the male individuals in MS patients was recorded as 1.1 ± 0.3(minimum = 1 and maximum = 3). In the female group, the average response to the EPT was 1.3 ± 0.6 (minimum = 1 and maximum = 2), respectively.

MS patients were asked about the trend of the disease and disease duration. Disease duration ranged from 12 to 156 months (Mean ± SD: 39.0 ± 51.5). Spearman correlation test was used to discover the relationship between duration of MS and teeth electrical stimulation thresholds. Although there was a positive relationship between these two variables, it was not statistically significant (*r* = 0.05 and *P* = 0.78).

## Discussion

In the present study, the responses of dental pulp nerve fibers to pulpal sensibility electrical test in MS patients were compared to healthy persons. There was a statistically significant difference between the two groups in pulpal responses.

There are some invisible symptoms in MS, like pain and depression. Clinicians should be notified of these invisible symptoms that could be more troubling than visible symptoms. They should ensure of adequate screening and treatment considerations in MS [12, 13].

According to the design of the Case-Control study, age, gender, and loss of evidence of trigeminal involvement were the same in both groups. Of course, all of the patients who entered the study were selected from a registered center for MS that was supervised by skillful neurologists. Patients fulfilled these criteria: relapsing-remitting type, no evidence of sensory/motor involvement in the trigeminal nerve, and no symptoms of trigeminal neuralgia in biography. It is worthy to be mentioned that in spite of different Disease-Modifying Treatment (DMT) in MS patients, all of them clinically were in controlled status since 1 year ago. This was confirmed by the related neurologist. Therefore, calibration of samples was completely achieved by this means.

EPT is used to determine the nerve perception threshold. Among vitality pulp tests, EPT with a little difference from cold test is the best one. EPT assesses the healthy Að nerve fibers in pulpal complex. Of course, the EPT is often unreliable in testing calcified teeth and old persons [14]. Regarding the point that MS as a neurodegenerative disorder could impact sensory nerves. Nerve conduction in dental pulp may be involved leading to the expression of inflammatory mediators and modifications of structural components of dental pulp. More studies have been done on peripheral nerve conduction in MS patients [15, 16]. Pogorzeski found that in 74% of MS patients at least one nerve is involved even in the absence of clinical symptoms [15]. EPT as the electrophysiological tester was selected. It is worthy to be mentioned that all participants should be free of any systemic disease with the side-effects of neuropathy. All of the above-mentioned inclusion criteria were considered. It should be considered that systemic doses of different types of analgesics can also alter the EPT responses. Also, caries or restorations, periodontal problems, history of trauma, orthodontic force, or unusual wear could complicate EPT results [17]. Hence, it was considered in the inclusion criteria. In Rendell’s study on sensory neuropathy of diabetic patients, it is shown that current perception thresholds have higher correlations with sensory clinical parameters than nerve conduction velocities [18].

Due to easier isolation, more accessible position, lower caries and point connection to adjacent teeth, and lower threshold to EPT, a central maxillary incisor was selected for the survey [19]. The age and gender of the two groups were matched to decrease patient bias. One-third incisal edge of teeth was selected as the optimal area for EPT evaluation. Some reasons for this idea are more concentration of neural components, less enamel thickness, direct pathway of dentinal tube, and low voltage required to stimulate [20].

Aging has a negative impact on the results of EPT [21]. Therefore, the mean age of participants in both groups was similar with no significant differences.

The method of this study was the same as that by Modaresi et al., but they compared diabetic patients with the Control group [22].

A study proved that trigeminal somatosensory evoked potentials were able to detect clinically silent lesions of MS [23]. The comparison of the means of the sensibility threshold of teeth elucidated a marked decrease in the MS group. The presence of a low threshold may be related to the pathological changes in MS patients [24]. Even in absence of clinical neuropathy, peripheral nerve excitability may be disturbed in MS patients. Lower electric pulp test thresholds in the MS group confirmed this [25].

Some dental literatures stated that electrophysiological abnormalities of MS were not associated with disease duration, disease course, and neurological disability [26, 27]. Gartzen et al. demonstrated subtle alteration on electrophysiological measurements in MS patients without hints for small fiber pathology [28]. In this study, although there was a positive relationship between disease duration and tooth response to electrical pulp test, it was not statically significant. The results of this study were consistent with past similar studies [15, 26]. Considering a mild positive relationship between disease duration and tooth pulp sensitivity, this hypothesis should be confirmed in future studies with a larger sample size.

According to the inclusion criteria, the duration of MS should be at least 1 year due to more severity of afferent fiber responses in the early stage [29]. There are plenty of studies discussing visual, brain stem auditory, and somatosensory evoked potentials in MS [15, 30], but attention to tooth pulp evoked potential is the novelty of this study nowadays. The interesting point of this study is the comparison of EPT threshold responses among MS patients to healthy persons [5, 30, 31]. Rendell compared the nerve conduction velocity in MS patients with healthy persons while this study presents a new approach by evaluation of EPT threshold of teeth has a new approach [32]. According to one study, the response to the electrical tests is a more sensitive indicator of the demyelination process than others [18]. Coggan in his study showed tongue somatosensory evoked potentials are an efficient method for evaluating the afferent trigeminal nerve in MS patients. He claimed it is more sensitive than MRI to detect early damage [33].

The current survey must be considered in light of certain limitations. Limited working hours of the specialized clinic of MS and outdated patients’ archived files were some of the limitations of the present study. Despite the limitations, the study is important and novel as a first step for designing future experiments. In MS patients, physiological changes to dental pulp could impact the normal response. Response threshold to electrical stimulation also decreased. This finding is not an indication of pulp vitality damage. It suggests that the results of electrical pulp testing need to be carefully interpreted and closely scrutinized in MS patients.

## Conclusion

Despite the limitations of this study, EPT could be applied as a recommended test for the evaluation of the trigeminal nerve response threshold. The MS patients without trigeminal neuralgia showed a significant reduced response to EPT in maxillary central incisors. The conclusion of this study should not be expanded without certain restraints.

## Data Availability

All data analyzed during this study are included in this published article. If any additional data/files may be obtained from the corresponding author on reasonable request.

## Abbreviations

MS: Multiple sclerosis
EPT: Electric pulp testing
